# Mortalin: Protein partners, biological impacts, pathological roles, and therapeutic opportunities

**DOI:** 10.3389/fcell.2023.1028519

**Published:** 2023-02-02

**Authors:** Niki Esfahanian, Cole D. Knoblich, Gaven A. Bowman, Khosrow Rezvani

**Affiliations:** Division of Basic Biomedical Sciences, Sanford School of Medicine, The University of South Dakota, Vermillion, SD, United States

**Keywords:** mortalin (HSPA9), cancer, protein partners, cellular localization, post-translational modification (PTM)

## Abstract

Mortalin (GRP75, HSPA9A), a heat shock protein (HSP), regulates a wide range of cellular processes, including cell survival, growth, and metabolism. The regulatory functions of mortalin are mediated through a diverse set of protein partners associated with different cellular compartments, which allows mortalin to perform critical functions under physiological conditions, including mitochondrial protein quality control. However, alteration of mortalin’s activities, its abnormal subcellular compartmentalization, and its protein partners turn mortalin into a disease-driving protein in different pathological conditions, including cancers. Here, mortalin’s contributions to tumorigenic pathways are explained. Pathology information based on mortalin’s RNA expression extracted from The Cancer Genome Atlas (TCGA) transcriptomic database indicates that mortalin has an independent prognostic value in common tumors, including lung, breast, and colorectal cancer (CRC). Subsequently, the binding partners of mortalin reported in different cellular models, from yeast to mammalian cells, and its regulation by post-translational modifications are discussed. Finally, we focus on colorectal cancer and discuss how mortalin and its tumorigenic downstream protein targets are regulated by a ubiquitin-like protein through the 26S proteasomal degradation machinery. A broader understanding of the function of mortalin and its positive and negative regulation in the formation and progression of human diseases, particularly cancer, is essential for developing new strategies to treat a diverse set of human diseases critically associated with dysregulated mortalin.

## Introduction

In the 1960s, Italian scientist Ferruccio Ritossa observed that cells could induce strong transcriptional activity in response to elevated temperatures (Ritossa, 1962; De Maio et al., 2012). This initial report led to the discovery of heat shock proteins (HSPs) several years later (Tissières et al., 1974). Besides stress-inducible HSPs, there are HSP genes that encode constitutively expressed members (Brown et al., 1993). HSPs have two fundamental roles in cells: Maintaining proper protein synthesis, and restoring cellular homeostasis in response to internal and external environmental stresses (Kumar et al., 2022). Constitutive and stress-responsive proteins are essential for properly folding proteins and preventing the aggregation of misfolded proteins, particularly as part of endoplasmic reticulum-associated degradation (ERAD) (Brodsky, 2007). Meanwhile, damaged proteins generated as a result of cellular physiological and pathological stressors are targeted by HSPs for a recovery process to return to their normal functions. Aside from protein folding, HSPs are necessary for cytoskeletal recognition and organization (Muranova et al., 2022) and play essential roles in the protection, integrity, and function of several intracellular organelles, including the mitochondria (Craig, 2018), Golgi apparatus (Wu et al., 2019), endoplasmic reticulum (Matsumura et al., 2013), lysosomes (Ingemann and Kirkegaard, 2014), and cell membrane (De Maio and Hightower, 2021). Alongside intracellular functions, HSPs are present in extracellular compartments covering several different roles (De Maio and Vazquez, 2013). Extracellular vesicles (EVs), including exosomes, are examples of extracellular compartments where HSPs demonstrate different biological impacts on cellular events. For example, extracellular HSPs play a critical role in cell/tissue homeostasis under normal conditions (Calderwood et al., 2021). Additionally, these extracellular HSPs (eHSPs) contribute to various diseases, including human cancers, by mediating inflammation and immunity (Lancaster and Febbraio, 2005; Wyciszkiewicz et al., 2019; Komarova et al., 2021; Li et al., 2022).

HSPs are historically divided into subfamilies according to their molecular weight, ranging from 10 to more than 100 kDa in molecular size, and are located in various cellular compartments (Jee, 2016). The 70-kDa subfamily (HSP70) is a family of proteins that function as molecular chaperones and are responsible for regulating the necessary housekeeping activities of the cell. They are essential for protein folding and refolding, protein modification, translocation, prevention of protein aggregation, protein degradation, formation and disassembly of protein complexes, regulation of apoptosis, and cell survival (Radons, 2016; Albakova et al., 2020). In addition to intracellular HSPs, extracellular HSP70 released from necrotic or stressed cells is immunogenic and contributes to the autoimmune reaction (Tukaj, 2020). Typically, HSP70 proteins require additional co-chaperones to aid their functions. These co-chaperones can positively or negatively interfere with the function of HSP70s (Laufen et al., 1999; Bhattacharya et al., 2020). A diverse set of HSP70 subfamily members are found in human cells, comprising the following 13 gene products: HSPA1A (HSP70-1), HSPA1B (HSP70-2), HSPA1L (hum70t), HSPA2 (heat-shock 70 kD protein-2), HSPA5 (Grp78, Bip, MIF2), HSPA6 (heat-shock 70 kD protein 6, or HSP70B′), HSPA7 (heat-shock 70 kD protein 7), HSPA8 (HSC70, HSC71, HSP71, HSP73), HSPA9 (Grp75, MOT, MOT2, PBP74, mot-2), HSPA12A (FLJ13874, KIAA0417), HSPA12B (RP23-32L15.1), HSPA13^b^ (Stch), and HSPA14 (HSP70-4, HSP70L1; (Kampinga et al., 2009). HSP70 subfamily members are found in various cellular compartments such as the cytoplasm, nucleus, lysosomes, endoplasmic reticulum, and mitochondria, indicating their essential biological functions in these organelles. Isoforms of HSP70 proteins have two major domains: An N-terminal ATPase domain, and a C-terminal substrate binding domain (SBD) connected by a hydrophobic linker domain (Zhuravleva and Gierasch, 2011). The C-terminal SBD domain consists of two subdomains, *α* and *β*, which act as a flexible lid and sandwich subdomain, respectively. However, structural differences determine their respective substrates, such as DnaK (prokaryotic HSP70) versus eukaryotic HSP70 (Zhang et al., 2014). While HSP70 subfamily proteins share the majority of structural features, mutations at their coding regions have allowed them to be targeted and retained to particular organelles (McCallister et al., 2015). One example is GRP75, referred to as mortalin hereafter, which has a 46-amino acid mitochondrial-targeting signal peptide at its N terminus (Dahlseid et al., 1994).

In addition to the basal function of HSPs in normal cells, HSPs have protective roles in cancerous cells. Intrinsic (elevation of oncoproteins during carcinogenesis) and extrinsic (low glucose, pH, and oxygen) stresses developed in cancer cells lead to overexpression of HSPs in diverse malignant tumors. High levels of HSPs correlate with more aggressive characteristics and chemoresistance (Ciocca and Calderwood, 2005). However, patterns of HSP expression are selectively determined by the stages of cancer as well as tissue types. Some examples are the biological impacts and prognostic values of HSPs (HSP27, HSP60, and HSP70) in prostate, breast, and colorectal cancers in both early and advanced cancers (Glaessgen et al., 2008; Javid et al., 2022; Sun et al., 2022; Tsai et al., 2022).

## Human mitochondrial Hsp70 (mortalin/mot-2)

Human mitochondrial Hsp70 (mortalin/mot-2/GRP75) is a heat uninducible HSP70 protein (Londono et al., 2012). Mortalin is similar to other HSPs, containing an N-terminus ATPase and SBD located at the C-terminus (Luo et al., 2010). Similar to other HSP70s, mortalin is shown to be highly expressed in several types of tumors. As a mitochondrial chaperone protein, mortalin plays a critical role in mitochondrial biogenesis. Mitochondrial mortalin mediates polypeptide chain translocation through the TOM/TIM translocase complexes, unfolds/folds nuclear proteins, and stabilizes translocated proteins. Additionally, mitochondrial mortalin is involved in biogenesis of iron-sulfur clusters in the matrix (Gambill et al., 1993; Voisine et al., 1999; Dutkiewicz et al., 2003), responds to glucose deprivation (Merrick et al., 1997), and protects against reactive oxygen species (ROS) (Tai-Nagara et al., 2014). While mortalin is a crucial mitochondrial protein, around 30% of mortalin in the cell is located in other cellular compartments such as the cytoplasm, endoplasmic reticulum (ER) (Szabadkai et al., 2006; D'Eletto et al., 2018; Liu et al., 2019b), and nuclear (Ryu et al., 2014) compartments and is circulated in blood (Priyanka and Seth, 2022). It has been shown that extracellular mortalin can have a vital survival role in cells attacked by complement-dependent cytotoxicity (CDC). Translocation of mitochondrial mortalin from mitochondria to the plasma membrane allows it to bind to the C8 and C9 complement components and inhibit C5b-9 assembly and stability, thus preventing induction of cell death mediated by complement-mediated lysis (Pilzer and Fishelson, 2005; Saar Ray et al., 2014; Tai-Nagara et al., 2014; Mazkereth et al., 2016). These locations, combined with a diverse set of protein partners, allow mortalin to positively and negatively regulate several pathways in both normal and pathological status.

## Mortalin and tumorigenic pathways

Overexpression of mortalin correlates with diminished patient survival in several forms of cancer, including early-stage non-small cell lung cancer (Sun et al., 2017), breast cancer (Zhang et al., 2021), hepatocellular carcinoma (Cheng et al., 2019), ovarian and cervical cancers (Putri et al., 2019; Xu et al., 2019), gastric cancer (Dai et al., 2021), and CRC (Rozenberg et al., 2013; Javid et al., 2022). In the context of CRC, overexpressed mortalin was observed in adenoma and colorectal adenocarcinomas (Dundas et al., 2005). In addition to the intracellular tumorigenic role of mortalin in above malignant tumors, exosomes derived from tumor cells of breast, ovarian, prostate, hepatic, gastric, colon, but not pancreatic carcinoma have high levels of mortalin expression compared to normal tissues (Huang et al., 2019).

In transformed cells, nuclear localization of p53 is essential for its tumor suppressor activity in terms of growth arrest and apoptosis (Shaulsky et al., 1991). Overexpression and enriched cytoplasmic mortalin in transformed cells allow mortalin’s SBD to bind to and interfere with the normal function of several proteins, including p53. One of the most well-studied oncogenic roles of mortalin is its ability to bind cytoplasmic p53, thereby sequestering it in the cytoplasm, inhibiting its nuclear localization and tumor suppressive properties (Gestl and Anne Bottger, 2012; Elwakeel, 2022). Meanwhile, it has been shown that mortalin-p53 interactions can be regulated with physiological or chemical stresses in a cancer-dependent manner (Lu et al., 2011). Delivery of mortalin-specific shRNA-expressing by adenoviruses can successfully supress the growth of breast cancer MCF7 xenograft tumors (Yoo et al., 2010). Supressing mortalin-p53 interaction by siRNA pharmacological tools such as MKT-077, Withaferin A, and mortalin protein suppressors leads to growth arrest and apoptosis, supporting the dominant p53 inhibitory role of mortalin in malignant tumors (Wadhwa et al., 2000; Wadhwa et al., 2002; Wadhwa et al., 2003a; Kaul et al., 2005; Yoo et al., 2010; Sane et al., 2014; Nigam et al., 2015).

Another protein that may explain the role of mortalin in tumorigenesis is CD151. CD151 is overexpressed in malignant tumor tissues and is found to be a driver of tumor progression and metastasis in several cancer types through the formation of tetraspanin CD151-enriched microdomains (Sadej et al., 2014). In hepatocellular carcinoma (HCC), mortalin was found to stabilize CD151-dependent tetraspanin-enriched microdomains and contribute to the progression of HCC (Liu et al., 2019a). Furthermore, altered centrosome amplification can lead to chromosomal instability, a highlighted feature of cancer cells. Ma et al. (2006) reported that overexpression of mortalin resulted in its localization to the centrosome and inhibition of p53-mediated suppression of centrosome duplication. Interestingly, a dominant mortalin inhibitor, UBXN2A, which interferes with mortalin-p53 interaction (Sane et al., 2016), is enriched in centrosomes [https://www.proteinatlas.org, UBXN2A’s subcellular section; (Thul et al., 2017)]. Another critical event in tumor progression is uncontrolled cell division. The Raf/MEK/ERK pathway is a signaling pathway responsible for cell survival. Studies in ovarian cancer demonstrated the expression of mortalin-induced Raf/MEK/ERK activation, highlighting mortalin’s regulatory role in the MAPK/ERK pathway, and tumor progression (Dundas et al., 2005; Hu et al., 2016). Further studies on the effect of mortalin on the Raf/MEK/ERK signaling pathway revealed that reduction of mortalin in MEK/ERK activated cells leads to elevation in the level of p21^CIP1^. The cyclin-dependent kinase inhibitor p21^CIP1^ triggers innate tumor suppressive mechanisms by the inhibition of cell growth. Interestingly, the mortalin-dependent regulation of p21^CIP1^ depends on MEK/ERK activity. The regulatory mechanism of mortalin on p21^CIP1^ is uniquely mediated by mortalin independent of p53 (Wu et al., 2013). Finally, a set of experiments in a rat adrenal pheochromocytoma (PC12) cell line under glucose deprivation revealed that mortalin can activate AKT in a PI3K-independent manner through the Raf/MEK/ERK signaling pathway. Activation of AKT and ERK by mortalin suppress conformational change in Bax, decrease cytochrome c release, and reduce apoptosis (Yang et al., 2011).

Besides tumorigenic functions in the cytoplasm, nuclear mortalin without the mitochondrial localization peptide was shown to increase the malignant properties of cancer cells, including cell proliferation, colony formation, motility, and tumor forming capacity in both *in vitro* and *in vivo* models. The nuclear mot-N mechanistically binds and supresses p53 function while functionally activating telomerase and heterogeneous ribonucleoprotein K (hnRNP-K) proteins to promote carcinogenesis (Ryu et al., 2014).

Lastly, overexpression of mortalin is associated with the enrichment of several cancer cells stemness markers, such as ABCG2, OCT-4, CD133, ALDH1, CD9, MRP1, and connexin. By increasing the population of stem cells, mortalin promotes the formation of spheroids (Yun et al., 2017). Elevation of cancer stem cells (CSCs) may explain the higher migration and drug resistance seen in the presence of overexpressed mortalin in cancer cells. Using breast cancer cells, Na et al. showed that overexpressed mortalin can downregulate epithelial markers E-cadherin (CDH1), CK8 (KRT8), and CK18 (KRT18). Simultaneously, overexpressed mortalin enhances mesenchymal markers, including vimentin, fibronectin, β-catenin, CK14, and hnRNP-K (Na et al., 2016). Mortalin’s multi-functional roles in tumorigenic pathways summarized in this section explain mortalin’s contributions to epithelial-to-mesenchymal transition (EMT), early tumor recurrence, tumor migration/invasion, stemness, angiogenesis, and tumor metastasis (Wadhwa et al., 2006; Yi et al., 2008; Chen et al., 2014; Na et al., 2016; Xu et al., 2020). Finally, a recent study in human breast cancer cells has shown that mortalin supports breast cancer stem cells and enhances EMT through activation of the Wnt/GSK3β/β-catenin signaling pathway (Wei et al., 2021).

## The prognostic role of mortalin in human cancer

**FIGURE 1:**
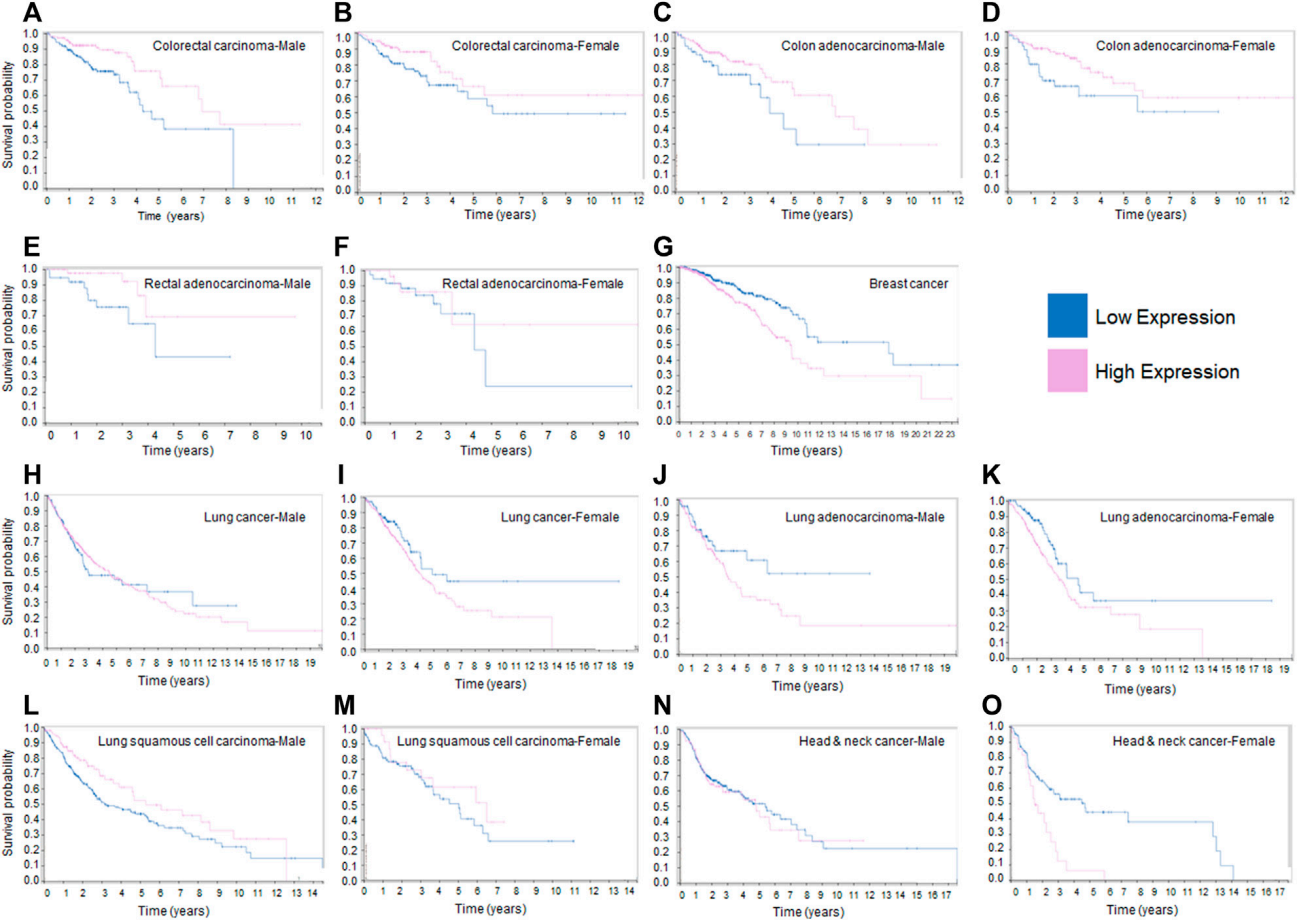
Kaplan-Meier survival curves of patients with different human cancers sorted according to expression levels of HSPA9/mortalin. **(A)**. In male patients with colorectal carcinoma, low expression of HSPA9 has a negative effect on survival. **(B)**. In female patients with colorectal carcinoma, low expression of HSPA9 has a negative effect on survival. **(C)**. In male patients with colon adenocarcinoma, low expression of HSPA9 has a negative effect on survival. **(D)**. In female patients with colon adenocarcinoma, low expression of HSPA9 has a negative effect on survival. **(E)**. In male patients with rectal adenocarcinoma, low expression of HSPA9 has a negative effect on survival. **(F)**. In female patients with rectal adenocarcinoma, low expression of HSPA9 has a negative effect on survival. **(G)**. In female patients with breast cancer, high expression of HSPA9 has a negative effect on survival. **(H)**. In male patients with lung cancer, HSPA9 expression levels have no effect on survival. **(I)**. In female patients with lung cancer, high expression of HSPA9 has a non-significant negative effect on survival. **(J)**. In male patients with lung adenocarcinoma, high expression of HSPA9 has a non-significant negative effect on survival. **(K)**. In female patients with lung adenocarcinoma, high expression of HSPA9 has a non-significant negative effect on survival. **(L)**. In male patients with lung squamous cell carcinoma, HSPA9 expression levels have no effect on survival. **(M)**. In female patients with lung squamous cell carcinoma, HSPA9 expression levels have no effect on survival. **(N)**. In male patients with head and neck cancer, HSPA9 expression levels have no effect on survival. **(O)**. In female patients with head and neck cancer, high expression of HSPA9 has a non-significant negative effect on survival. The prognostic value of mortalin RNA expression in different cancers illustrated in this figure were downloaded from the Human Protein Atlas: https://www.proteinatlas.org/ENSG00000113013-HSPA9/pathology.

Based on the tumorigenic activities of mortalin in malignant tumors discussed above, we decided to investigate the survival curves of cancer patients by assessing the RNA expression level of HSPA9/mortalin using The Cancer Genome Atlas (TCGA) available in the human protein atlas database (Uhlen et al., 2017). Figure 1 shows that mortalin is a prognostic factor in breast and colorectal cancers, the second and third most common cancers, respectively (Bray et al., 2018). However, in colorectal cancer (carcinoma or adenocarcinoma), low expression of mortalin is an adverse prognostic factor (Figures 1A–F), whereas in breast cancer low expression of mortalin is a favorable prognostic factor (Figure 1G). The adverse effect of low RNA expression of mortalin in colon and rectal adenocarcinoma has no correlation with expression of mortalin protein previously reported (Dundas et al., 2005). A part of this discrepancy is that the RNA and protein levels of specific genes are not correlated in tumor tissues. The dysregulated transcription machinery, microRNAs and post-translational modifications in transforming cells can cause uncorrelated RNA-protein relations for specific genes as previously described (Kosti et al., 2016). Interestingly, mortalin’s impact on prognosis differs between rectal and colon adenocarcinoma (Figures 1C, D versus Figures 1E, F), as these two tissues are different in terms of pathological features (Hemminki et al., 2010) and survival rate (Lee et al., 2013).

In lung cancer, mortalin is not found to be a significant prognostic factor. However, in women, a high expression of mortalin appears to be an adverse prognostic factor (Figures 1H, I). When categorizing lung cancer into adenocarcinoma and squamous cell carcinoma, a high expression of mortalin is found to have an adverse effect on survival in the former (Figures 1J, K) but no significant impact in the latter (Figures 1L, M). Finally, in head and neck cancer, while mortalin is not a prognostic factor in male patients, there appears to be a trend towards higher expression being an adverse prognostic factor in female patients (Figures 1N, O). The RNA-sequencing data in Figure 1, generated from TCGA transcriptome database, suggests mortalin’s tumorigenic or tumor suppressive function depends on the specific cancer tissues. Furthermore, this data suggests sex is another factor that can determine the function of mortalin in malignant cells, as previously described (Li et al., 2018). However, it has been shown that the gene-expression values derived by TCGA pipeline can vary considerably across biological replicates (Rahman et al., 2015). Further studies are necessary to determine the role of mortalin, particularly at the protein level, in a tissue-specific, cell-type-specific, and stage-dependent manner in tumorigenesis and the organization of oncogenic signaling pathways. Western blot experiments revealed that human CRC and breast tumors have significantly elevated levels of mortalin and showed that patients with overexpressed mortalin are commonly diagnosed with a higher grade and stage of tumors, indicating lower chance of survival (Abdullah et al., 2015a). Beyond the expression levels of mortalin, its binding partners and post-translational modifications are other critical factors in mortalin’s functions in malignant tumors (Schneider et al., 2017).

## Mortalin binding partners

Published research articles indicate mortalin crosstalks with a diverse protein network. A variety of biochemical, biological, and computational methods with different levels of selectivity and specificity have been used to investigate mortalin’s interaction partners and their biological mechanisms in diverse types of *in vitro* and *in vivo* models. Based on the interaction dynamics, interactions between mortalin and its partners can be weak or strong, transient or stable, non-permanent or permanent. Several molecular and cellular factors such as kinetics, thermodynamics, stoichiometry, and cofactors can affect mortalin’s interaction with its partners in a spatiotemporal manner (Lobingier et al., 2017). We decided to list several of mortalin’s partners reported in the last decade in the literature. While discussion of mortalin’s protein partners can provide a valuable resource for future studies, some of these protein-protein interactions and their roles at the molecular and cellular levels needs further confirmation.

Mortalin has several different protein partners crucial for its function in the mitochondria. The main binding partners of the HSP70 subfamiliy is comprised of two co-chaperones: J-domain proteins, and nucleotide exchange factors (NEFs). These two co-chaperones are essential for the protein-folding function of HSP70 proteins, as J-protein stimulates ATPase activity, and NEFs promote the release of nucleotides. In eukaryotic cells, NEFs have a vital role in protein folding and import as well as protein quality control (Bracher and Verghese, 2015). Different cellular compartments have various combinations of these two proteins, allowing for specialized functions of HSP70 proteins (in this case, mortalin) in the mitochondria (Moro and Muga, 2006; Marszalek, 2016).

P66shc protein mediates insulin resistance, promotes apoptosis, and controls the intracellular redox balance (Biondi et al., 2022; Haslem et al., 2022). In HCC, downregulated p66shc is associated with better survival (Fasolato et al., 2021). Under normal conditions, p66Shc is tightly controlled to avoid unnecessary induction of apoptotic signals originated in the mitochondria (Orsini et al., 2006). In the absence of cell stressors, a fraction of cytosolic p66Shc localizes within the mitochondrial matrix to form a complex with mortalin. Apoptotic events such as ultraviolet radiation induce the dissociation of p66Shc from mortalin and the release of free p66Shc. The dissociated p66Shc can enhance the mitochondrial pathway of apoptosis by inducing mitochondrial damage (Orsini et al., 2004).

A mitochondrial zinc finger motif heat shock protein, Zim17, binds to mortalin inside the mitochondria and is involved in several critical mitochondrial functions. The TimI5/Ziml7 complex cooperates with mortalin to facilitate the transfer of unfolded proteins into the mitochondrial matrix (Yamamoto et al., 2005). Studies in yeast have shown that the depletion of Zim17 suppresses mitochondrial protein import, interferes with Fe/S protein biogenesis, and leads to aggregation of mortalin in the matrix (Sanjuán Szklarz et al., 2005). Human Hsp70 escort protein (Hep) is a human Zim17 ortholog. Uniquely, mammalian Zim17 (Hep) can facilitate the ATPase activity of human mortalin, and it prevents the aggregation of unfolded target proteins (Goswami et al., 2010).

In addition to mortalin, HSP60 is a vital chaperone for unfolded proteins in the mitochondria (Cheng et al., 1989). The two proteins work similarly to assist protein folding in the mitochondrial matrix but are also found in other subcellular locations with similar functions (Deocaris et al., 2006). An interaction between the N-terminal domain of mortalin and HSP60 has also been demonstrated in both *in vitro* and *in vivo* conditions (Wadhwa et al., 2005). Malfunction of mortalin and its HSP60 partner leads to the accumulation of unfolded aggregated proteins in response to stress, aging, or disease (Iosefson et al., 2012). A more recent study revealed that proteasomal degradation of mortalin in the cytoplasmic compartment leads to reduction of mortalin’s partner, HSP60, in mitochondria. This finding indicates that the presence of mortalin is essential for mortalin-HSP60 protein quality machinery in the mitochondria (Sane et al., 2018). Another functional binding partner for mortalin’s disaggregating mechanism in the mitochondria is Tid1, a known HSP40 protein. Therefore, the mortalin-TID1 complex can potentially respond to aggregation during cellular stress. More importantly, the efficient function of mortalin-Tid1 can be a therapeutic target to slow or reverse the aggregation of toxic proteins in pathological conditions such as neurodegenerative diseases (Khan et al., 2020).

Accumulating evidence indicates that mitochondria play critical roles in the cancer cell metastatic cascade by promoting invasion, intravasation, and extravasation. A dysregulated mitochondrial bioenergetic state due to the abnormal function of mitochondrial protein quality control supports cancer cells in their divergent microenvironments during tumor cell migration and invasion (Guha et al., 2014; Cui et al., 2017; Han et al., 2018; Lin et al., 2018; Porporato et al., 2018; Emmings et al., 2019). The lack of knowledge regarding regulatory pathways abused by mitochondria in cancer and the unexamined protein complexes within *in vivo* models limit the number of therapeutic approaches that specifically target mitochondria in cancer cells. Finding novel pathways such as the mortalin-HSP60 axis and its regulatory elements can be a new platform for a therapeutic approach that not only effectively represses primary tumor progression but also suppresses the development of metastatic characteristics interfering with overactive mitochondria.

Interactions between the mitochondria and the ER allow for resident ER proteins to interact with mortalin. Two important proteins that cooperate with mortalin are the inositol 1,4,5 triphosphate receptor (IP3R) and voltage-dependent anion channel 1 (VDAC1) (Xu et al., 2018). Together, these three proteins assist with Ca^2+^ transfer from the ER to mitochondria, with mortalin acting as the bridge between IP3R and VDAC1 (Xu et al., 2018). Transglutaminase type 2 (TG2) is a mortalin’s partner in mitochondria-associated membranes (MAMs) that regulate ER-mitochondria contact sites. Reduction of TG2-mortalin increases interaction between IP3R3 and mortalin. TG2 and its binding to mortalin can control ER-mitochondrial Ca^2+^ flux and protein expression (D'Eletto et al., 2018). A study focused on early onset Parkinson’s disease (PD) has shown that DJ-1 interacts with the IP3R-mortalin-VDAC complex and is essential for proper communication between the mitochondria and ER (Liu et al., 2019b).

Another key partner of mortalin is apoptosis-inducing factor (AIF), which plays a major role in the caspase-independent apoptotic pathway (Liu et al., 2006). It has been shown that mortalin interacts with AIF to mediate its binding to the outer mitochondrial membrane (OMM). In addition, chemotherapeutic agents decrease AIF-mortalin interaction, resulting in the dissociation of AIF from the OMM and, consequently, the induction of apoptosis (Fadeeva et al., 2018). Further studies are needed to determine whether inhibition of AIF-mortalin interaction, dissociation of AIF from the OMM, and subsequent apoptosis can potentially turn into a therapeutic strategy in cancer cells with high levels of mortalin, such as glioma patients.

Outside of the mitochondria, mortalin’s binding partners are varied. *In vivo* studies demonstrated that GRP94, an HSP90-like chaperone of the endoplasmic reticulum, is a binding partner (Takano et al., 2001; Marzec et al., 2012). Similar to its role in the mitochondrial import of unfolded proteins, mortalin mediates a similar role in intracellular import in the cytoplasm (Mizukoshi et al., 1999). Mortalin binds fibroblast growth factor 1 (FGF1), allowing for its uptake and appropriate path to its target organelle in the cell (Mizukoshi et al., 1999). The IL-1 receptor is similarly internalized through ATP hydrolysis by mortalin, leading to downstream cytokine pathways in the cell (Sacht et al., 1999). Additionally, a set of cytoplasmic proteins interact with mortalin and interfere with the formation of a mortalin-p53 complex in the cytoplasm. One such example is DAB2IP, which competitively binds mortalin, effectively preventing the degradation of p53 (Feng et al., 2022).

A set of studies reported by Wadhwa et al. (2003b) has shown that interaction between mortalin and mevalonate pyrophosphate decarboxylase (MPD) can result in reduced levels of Ras and phosphorylated ERK2. Since oncogenic Ras facilitates cancer cell proliferation, inhibition by mortalin suggests a safeguard mechanism. This study indicates that mortalin affects cell proliferation by more than one mechanism, which can be exchanged during cancer cell transformation in a cellular context matter. Current evidence strongly supports mortalin’s functions as a proliferation-controlling complex under normal and pathological conditions. Future mechanistic studies will determine whether mortalin is a promising therapeutic target in selected malignant tumors.

Using a set of protein-protein assays, Hansen et al. (2015) reported that mortalin binds to the CREC family, which is a set of multiple EF-hand Ca^2+^-binding proteins. Reticulocalbin, ERC-55 and its splice variants, reticulocalbin-3, Cab45 and its splice variants, and calumenin are all in the CREC family. Members of the CREC family bind and regulate a number of proteins in the ER and sarcoplasmic reticulum (SR). By binding to mortalin, calumenin and reticulocalbin allow CREC proteins to contribute to diverse pathways, including chaperone activity, cell proliferation, transformation, and cellular aging (Hansen et al., 2015).

It has been shown that mitochondrial proteolytic stress induced by loss of mortalin function can be rescued by Parkin and PINK1 protein. To rescue loss of mortalin phenotypes, Parkin and PINK1 increase lysosomal-mediated mitochondrial clearance and facilitate autophagic machinery (Burbulla et al., 2014). The current evidence indicates that PINK1 can function as either a pro- or an anti-tumorigenic protein in different tumors, showing a duality dependent on context (Wang et al., 2022). PINK1 regulates cell survival, stress resistance, mitochondrial homeostasis, and the cell cycle, all of which are key signaling pathways misused by cancer cells during tumor initiation and progression. Mechanistically, PINK1 interferes with the PI3-kinase/Akt/mTOR signaling pathway, resulting in critical changes in mitochondrial and metabolic functions. These are essential for cancer survival, growth, stress resistance, and the cell cycle (Rakovic et al., 2011). Mechanistic crosstalk between PINK1 and mortalin requires further study, as PINK1-mortalin interaction could be a potential target for new therapeutic approaches in certain diseases, including Parkinson’s disease and malignant tumors.

Cancer cells express a diverse set of regulatory proteins to block the C5b-9 membrane attack complex (MAC) which induces cell death (Fishelson et al., 2003). By shedding of membrane vesicles loaded with complement MAC, mortalin can protects cancer cells from complement-mediated lysis (Fishelson et al., 2003). Rozenberg et al. has shown that Hsp90 can additionally protect cells from the complement-dependent cytotoxicity (CDC) by inhibiting C5b-9 assembly and/or stability at the plasma membrane. Interestingly, the complement activation can lead to direct binding of Hsp90 to mortalin. Formation of Hsp90–mortalin heterocomplexes in the presence of activated C5b-9 can generate an efficient protection against C5b-9 (Rozenberg et al., 2018).

## Post-translational modification and regulation of mortalin

Post-translational modifications (PTMs) of mortalin can affect its binding partners and specific function. For instance, phosphorylation of tyrosine residues augmented the interaction between mortalin and fibroblast growth factor 1 in the late G1 phase, emphasizing the importance of mortalin post-translational modifications in the regulation of the cell cycle (Mizukoshi et al., 1999). Additional PTMs include acetylation, oxidation, and ubiquitination (Deocaris et al., 2008) but further studies are needed to understand these PTMs and their function at the cellular level. Subsequently, selective activation or inhibition of mortalin’s PTMs by therapeutic interventions may be a possible avenue of therapy, preventing the downstream cancer-promoting signaling cascades.

Normal evolutionary mammalian cells have developed several regulatory mechanisms to avoid malignant transformation of normal cells in a tissue-dependent manner. Anti-tumorigenic mechanisms mediated by these tumor suppressor proteins and their associated proteins can target key proteins involved in tumorigenic pathways and stop or slow tumor progression. One protein that promotes the degradation of mortalin is UBXN2A (Sane et al., 2018). Similar to other ubiquitin-like proteins, UBXN2A targets a specific set of substrates in a cell-dependent manner. UBXN2A has a dual effect on the mortalin tumorigenic pathway. By binding to mortalin’s binding pocket (Sane et al., 2016), UBXN2A blocks mortalin’s interaction with its substrates, including the p53 tumor suppressor protein (Abdullah et al., 2015b). Consequently, UBXN2A recruits the CHIP E3 ubiquitin ligase and facilitates the ubiquitination of mortalin. Ubiquitination of the carboxy-terminal of mortalin leads to its proteasomal degradation (Sane et al., 2018). Future studies, including ongoing projects in our group, will determine whether UBXN2A-dependent degradation of mortalin alters the fate of mortalin protein partners and improves cancer cell response to traditional anti-cancer therapies. Besides its tumor suppressor function, UBXN2A plays a regulatory role in the nervous system (Rezvani et al., 2009; Teng et al., 2015). The mechanistic relation between UBXN2A and mortalin in the nervous system is another potential future study that can uncover the function of mortalin in neurodegenerative diseases.

The ubiquitin and ubiquitin-like proteins are involved in various pathologies such as inflammatory and neurodegenerative diseases as well as cancer (Hwang et al., 2022). It has been shown that many of these disease-related pathways can be modified and regulated by different types of modifications. The ubiquitin-like proteins can determine the fate of selected proteins using a single or combination of mono-ubiquitination, poly-ubiquitination, sumoylation, and neddylation. Therefore, understanding the mechanisms behind the upregulation and downregulation of mortalin by the ubiquitin-proteasome pathway can potentially lead to more specific anti-mortalin drugs.

## Conclusion and future work

**FIGURE 2:**
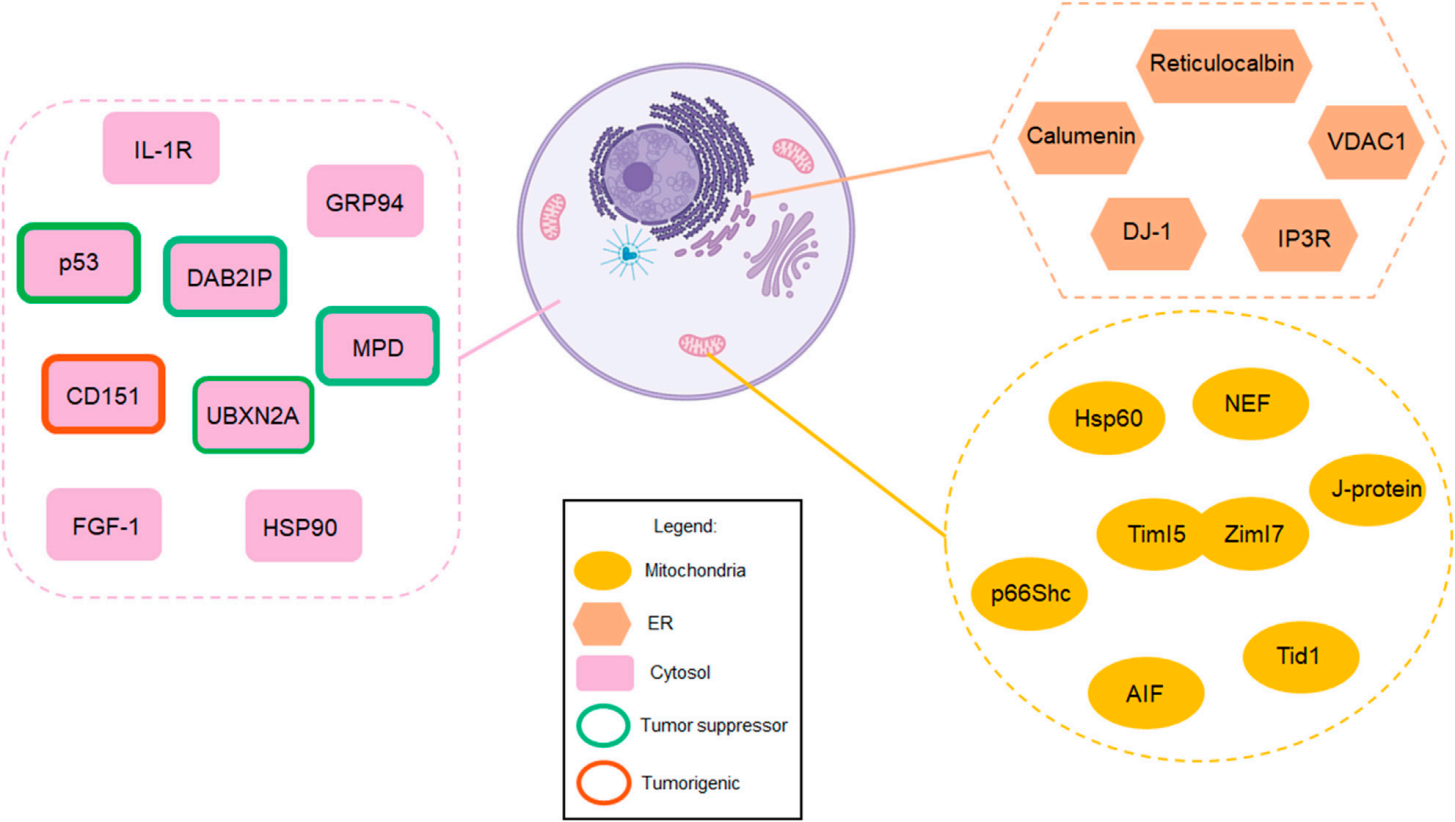
Mortalin acts as a “double agent,” playing contrasting roles in normal cells versus cancer cells. This figure summarizes mortalin’s partners in different subcellular compartments, including mitochondria, the cytosol, and the endoplasmic reticulum (created with BioRender.com). While mortalin’s functions are essential for normal cells, its interaction partners allow mortalin to become a poor prognostic factor in human cancer. Understanding these two different faces of mortalin will enable the design of anti-mortalin drugs that regulate its tumorigenic pathways in cancer cells while its physiological functions in normal cells remain intact. These partners have different binding affinities to mortalin, and their interactions are conditional on the cellular context. Therefore, future studies will determine the biological and pathological impact of these interactions in a tissue-dependent manner.

Mortalin, as a mitochondrial HSP, controls the protein quality process in the mitochondrial matrix under physiological conditions. However, overexpression and abnormal cellular compartmentalization turn this HSP into a driver of tumor progression in diverse types of malignant tumors. Mortalin’s diverse network of interaction partners enable it to positively or negatively regulate several pathways involved in cancer cell initiation and progression. In addition, mortalin promotes cancer cell migration and invasion, two key elements during tumor metastasis. As an element of the hallmarks of cancer, metastasis is the most significant factor in cancer-related deaths. Understanding the biological impact of mortalin during cancer progression and its tumorigenic mechanisms will introduce new paradigms to the study of tumor growth and metastasis. Certainly, mortalin’s partners and subcellular localizations are two key elements in mortalin’s tumorigenic functions. Additionally, the tumorigenic function of extracellular mortalin and its interaction with the tumor microenvironment is another unstudied area. Investigating the spatial and temporal function of mortalin in a cancer-dependent manner will improve the understanding of the pathological principle of mortalin, resulting in more effective and safer targeted therapies. Figure 2 and Supplementary Table S1 in this review list a set of mortalin’s partners reported in different *in vitro* and *in vivo* models using diverse methods and analytical approaches. These partners have different binding affinities to mortalin, and their interactions are conditional on the cellular context. Summarization of mortalin partners, and furthermore their direct and indirect relation with mortalin, illustrated in Figure 2, provides a helpful guideline for researchers who wish to conduct further studies on mortalin or mortalin partners. Understanding the biological mechanisms of pro-cancer proteins such as mortalin is crucial to opening therapeutic windows for successful novel interventions. The background and recent advancements covered in this review article indicate a safe anti-mortalin treatment can become an effective targeted therapy in a set of cancers with abnormal mortalin.
