# Supplementary material for: Mortalin: Protein partners, biological impacts, pathological roles, and therapeutic opportunities
Source: Front Cell Dev Biol. 2023 Feb 2;11:1028519. doi: 10.3389/fcell.2023.1028519 (PMC9932541; doi:10.3389/fcell.2023.1028519)
Supplement: Supplementary file 1 [file DataSheet1.PDF]

Supplementary materials:

Partners	Cellular Compartment	Reference (s)
HSP90	Cytoplasm	PMID: 29396434
UBXN2A	Cytoplasm	PMID: 24625977
IL-1R	Cytoplasm	PMID: 10221157
GRP94	Cytoplasm	PMID: 11439088
p53	Cytoplasm	PMID: 22683628<br>PMID: 16176931<br>PMID: 35493098
DAB2IP	Cytoplasm	PMID: 35150809
MPD	Cytoplasm	PMID: 12646231
CD151	Cytoplasm	PMID: 31772652
FGF-1	Cytoplasm	PMID: 10510314
Reticulocalbin	ER	PMID: 26161649
Calumenin	ER	PMID: 26161649
DJ-1	ER	PMID: 31767755
IP3R	ER	PMID: 29907098
VDAC1	ER	PMID: 29907098
HSP60	Mitochondria	PMID: 15957980
NEF	Mitochondria	PMID: 28132457<br>PMID: 16600294
p66Shc	Mitochondria	PMID: 15078873
TimI5-ZimI7	Mitochondria	PMID: 15642367
J-protein	Mitochondria	PMID: 28132457<br>PMID: 16600294
Tid1	Mitochondria	PMID: 33100870
AIF	Mitochondria	PMID: 16728972

Table 1: Mortalin protein partners in the endoplasmic reticulum (ER), mitochondria, and cytoplasm.
